# Surface properties of alkylsilane treated date palm fiber

**DOI:** 10.1038/s41598-022-13615-1

**Published:** 2022-06-13

**Authors:** Helanka J. Perera, Anjali Goyal, Saeed M. Alhassan

**Affiliations:** 1 Maths and Natural Science, Abu Dhabi Women’s Campus, Higher Colleges of Technology, Abu Dhabi, United Arab Emirates; 2 Department of Chemical Engineering, Khalifa University, PO Box 127788, Abu Dhabi, United Arab Emirates

**Keywords:** Plant sciences, Chemistry, Materials science

## Abstract

The present work focuses on investigating the effect of *non-fluoro short-chain alkylsilane* treatment on the surface characteristic of date palm (*Phoenix dactylifera*) fiber. Raw date palm fiber (DPF) was treated with octylsilane and the surface properties of treated fiber was investigated using thermogravimetric analysis (TGA), fourier transform infrared (FTIR) spectroscopy, scanning electron microscopy (SEM), contact angle analysis and X-ray diffraction (XRD) on configuring the thermal stability, chemical structures and surface properties (morphology, hydrophobicity and crystallinity). The decomposition temperature of 75% mass loss raw and treated DPF, the onset of temperatures were increased from 464 to 560 °C with the introduction of alkylsilane. Hydrophobicity and crystallinity index of the DPF fibers were increased from 66.8° to 116° and 31 to 41, introducing octylsilane to raw DPF. The SEM and XRD experimental results showed that the octylsilane treatment could effectively increase the pore size and crystallinity index as an indication of the removal of non-crystalline cellulosic materials from DPFs. Thermal stability, hydrophobicity and crystallinity of the fibers increased on DFP after alkylsilane treatment. The results indicated that alkylsilane-treated DPFs were a suitable reinforcing substitute for hydrophobic polymer composite.

## Introduction

Over the past decades, polymer composites incorporated natural renewable fiber materials have taken the attention of researchers due to their lightweight (which makes composites lighter), low cost, bio-renewable character, biodegradable and resistant to deforestation^1–3^. Many studies have demonstrated that natural fibers have replaced conventional synthetic fibers as reinforcing material on polymer composites^1–8^. Many literature studies were carried out on different plant fibers, including cotton, rice husk, wheat straw, oil palm, bagasse jute, coir and date palm^7,8^.

In Gulf countries, the date palm is one of the most widespread plants among all other trees. The United Arab Emirates (UAE) is home to the most date palms globally. It is said to have 40 million date palm trees and at least 200 cultivars, 68 of which are commercially valuable^9^. The UAE has 16,342,190 productive date palms in 2006, which produced 757,600 tons of dates^10^. The United Arab Emirates was just named the world's leading date palm cultivator, with 42 million trees, as revealed on March 15, 2009. Due to the more considerable amount of date palm production, a significant amount of waste is generated annually and burned directly in an open field, which causes damage to the environment and humans. Considering all problems caused by waste materials can be used as renewable materials for economically valuable product synthesis, such as crates, basketry, rope and furniture. Last decade, date palm fiber (DPF) as a reinforcing agent on polymer composite is one of the interesting topics in research^11–14^. However, several downsides were questioned while using these natural plant fibers as a reinforcing material. Due to the water absorption tendency, poor compatibility between hydrophobic matrix and fibers is considered the leading cause because of three main plant fiber components: cellulose, hemicellulose and lignin, which are responsible for the hydrophilicity in the fibers. But this drawback can be suppressed by modification of fiber surface through chemical treatment that switchover to hydrophobic behaviors and promote adhesion between polymeric matrix and reinforcing materials^15^.

According to the literature, numerous ways have been investigated for surface modification of fibers^16,17^, with alkali^18^, silane^19,20^, water-repelling agents^21^ and peroxide treatment^22^ being a couple of treatment methods to modify the natural fibers. However, the silanization method is one of the more effective chemical methods for introducing surface hydrophobicity^23–25^. Research carried out by Mukhtar et al.^26^ reported the impact of alkali and sodium carbonates used to treat the sugar palm fibers enhanced crystallinity, thermal stability and surface roughness compared to untreated fibers. Moreover, surface modification for DFPs carried out by Elbadry et al.^16^ by using heat and chemical treatment in the furnace at 100 °C for 1 h and 1% NaOH at 100 °C for 1 h, respectively, able to clean the wax and fatty substances on fiber surface while leading on the improved mechanical performance of fibers. The optimal alkali concentration for surface treatment of DPF study carried out by Oushabi et al.^11^ reported usage of 5% NaOH in an aqueous solution enhances the thermal resistance of fibers and interfacial properties between interfacial properties fibers and polyurethane matrix. The same group^12^ extended their work with 5% NaOH treated DPFs further modified with silane coupling agents (3-mercaptopropyltrimethoxysilane and 3-aminopropyltrimethoxysilane) in various concentrations to improve interface bonding between the fibers and the matrix. In a similar trend for surface modification of DFPs, Perera et al.^19^, our recent published work, highlight a comparison of fluoro and non-fluorosilane coupling agents on improvements in the structural and thermal properties of raw DPF liquid phase silanization technique.

In this study, *DPFs were chemically treated in the presence of non-fluoro short-chain alkyl silane (octyltrichlorosilane) to create a hydrophobic surface to assess their feasibility as a polymer composite reinforcing material*. Fourier transform infrared spectroscopy (FTIR), scanning electron microscope (SEM), thermal gravimetric analysis (TGA), contact angle and X-ray diffraction (XRD) analysis were used to investigate the chemical structure, morphology and physical properties (thermal, hydrophobicity and crystallinity) of raw and treated DPFs. This study aims to provide a systematic overview of how non-fluoro short-chain alkylsilane can be used to alter the surface and thermal properties of DPF.

## Experimental section

### Materials

**Figure 1:**
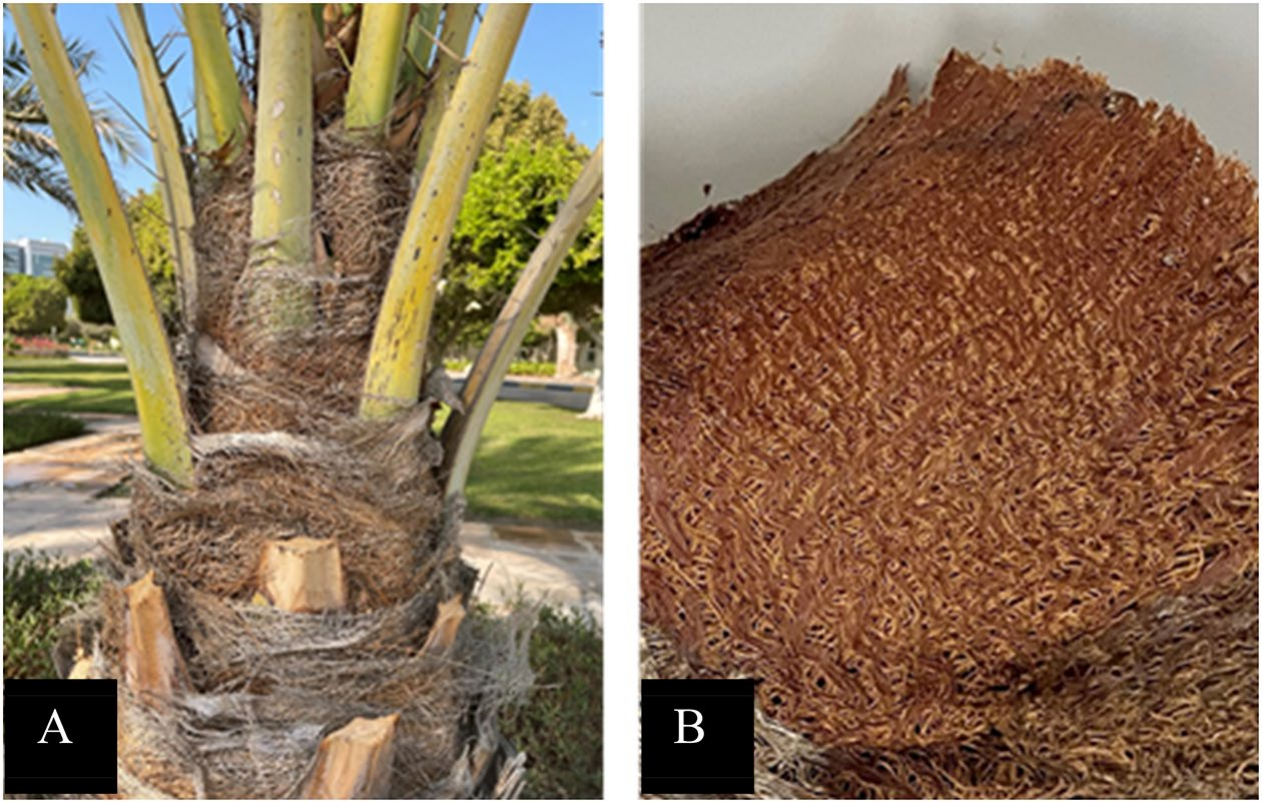
(**A**) DPF tree, (**B**) DPF meshes from date palm tree.

Octyltrichlorosilane (C8) was obtained from SigmaAldrich (St. Louis, MO, USA). Hexane was from Pharmco-Aaper (Brookfield, CT, USA). All chemicals were used as received. The raw date palm fiber (*Phoenix dactylifera*) meshes were collected from the Abu Dhabi Campus from Abu Dhabi, UAE (Fig. 1A). Date palm meshes were removed from the stem and sealed in polyethylene bags until the experiments were conducted (Fig. 1B). These DPF meshes were manually separated into single fibers and washed with distilled water to remove dust particles, sand and impurities. Cleaned fibers were dry for 24 h at room temperature.

## Methods

### Preparation of the treated date palm fibers

DPF (1 g) was cleaned and reacted with 1 g of C8 in glass vials with 250 mL of hexane to cover the DPF completely. The silane reaction was carried out in a shaker for 4 h at 50 °C and 200 rpm. After shaking, treated DPFs were rinsed three times with 100 mL hexane to remove the unreacted C8 coupling agent. After that, the materials were maintained at room temperature until the characterizations were performed. All the plant experiments were in compliance with relevant institutional, national and international guidelines and legislation.

### Measurements and characterization

#### Thermo-gravimetric analysis (TGA)

TGA was performed to identify the degradation characteristics of the C8 treated fiber with the raw DPF. Hereafter, the grafted amount of C8 on the treated DPF was quantified using a TA Instruments, Model SDT 650 Thermogravimetric Analyzer (TA Instruments, New Castle, DE, USA). The treated DPF and raw DPF samples were heated from 20 to 900 °C with a heating rate of 20 °C/min in high purity nitrogen gas. The TGA thermograms were measured by running three samples from three different places of the untreated/treated DPF samples.

#### Fourier transform infrared (FTIR) spectroscopy

FTIR spectra were taken using a Perkin Elmer Frontier FTIR spectrometer (PerkinElmer Genetics Inc., Waltham, MA, USA). The scanning range was from 600 to 4000 cm^−1^ with a spectral resolution of 4 cm^−1^ and 32 scans. The three samples from three different places of the raw/treated DPF samples were run during FTIR analysis.

#### Scanning electron microscopy (SEM)

The surface morphology was carried out by scanning electron microscopy (SEM) using FEG Quanta 250 (FEI Company, Hillsboro, OR, USA) instrument of the raw and C8 treated DPFs under high vacuum mode operated at an acceleration voltage of 5 kV a working distance of about 10 mm. For SEM studies, each sample was attached to double-sided carbon adhesive tape on the top of an aluminum stud. The samples were then made conductive by the sputtering of Au/Pd.

#### Contact angle analysis

**Figure 2:**
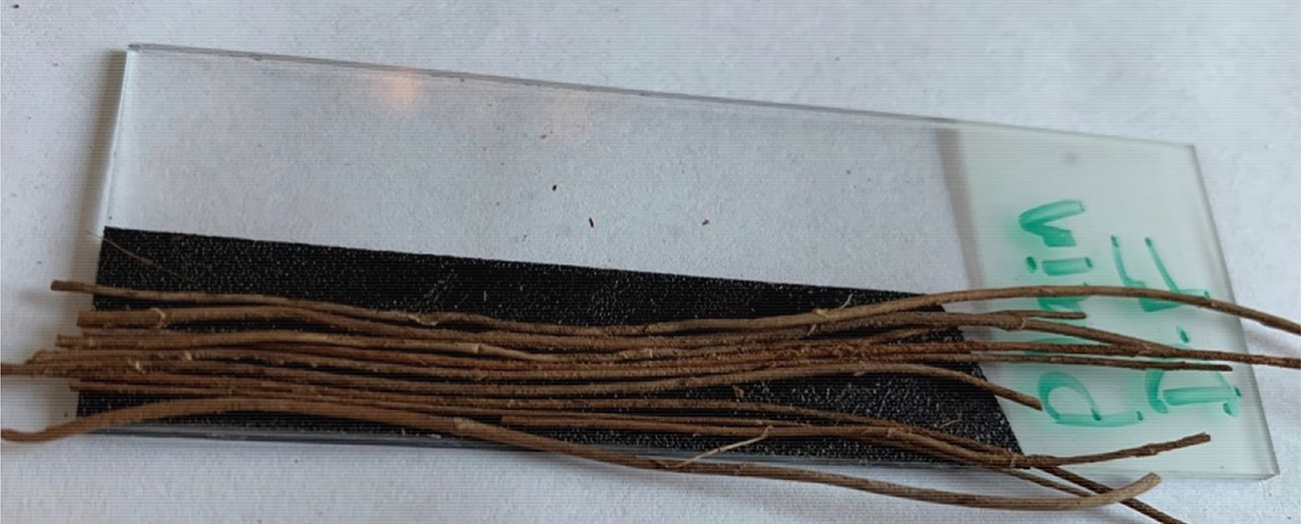
Fiber arrangement on a glass slide for contact angle measurement.

For contact angle measurements, the raw and C8 treated DPF samples were prepared on a glass slide by placing fibers close to each other with the help of double-sided sticky tape, as shown in Fig. 2. Water contact angle measurements were then performed using the static drop method at room temperature using KRÜSS DSA25 Series (KRÜSS Scientific Instruments, Inc., Matthews, NC, USA). Deionized water was used as a probe liquid (0.3 µl dispense volume) at a frequency of 20 in a time interval of 3000 ms. Ten images from different locations on the surface were taken for each sample, with the average reported for the contact angle.

#### X-ray diffraction (XRD)

Diffraction (XRD) patterns were collected using X’Pert PRO powder diffractometer (Cu-Ka radiation 1.5406 Å, 45 kV, 40 mA) in the range of 5°–80°, 2θ scale. The empirical method was used to obtain the crystallinity index (CI) of the samples^27^, as shown in Eq. (1):

1 $$CI=\left(\frac{{I}_{cr}-{I}_{am}}{{I}_{cr}}\right) \times 100$$

where I_cr_, and I_am_ represent the crystalline intensity and amorphous intensity at an angle (2θ). I_cr_ is the crystalline peak corresponding to the intensity of approximately 23° and I_am_ is the amorphous peak corresponding to the intensity of approximately 19°. The XRD curves were measured by running three samples from three different places of the untreated/treated DPF samples.

## Results and discussion

### Thermogravimetric analysis (TGA)

**Figure 3:**
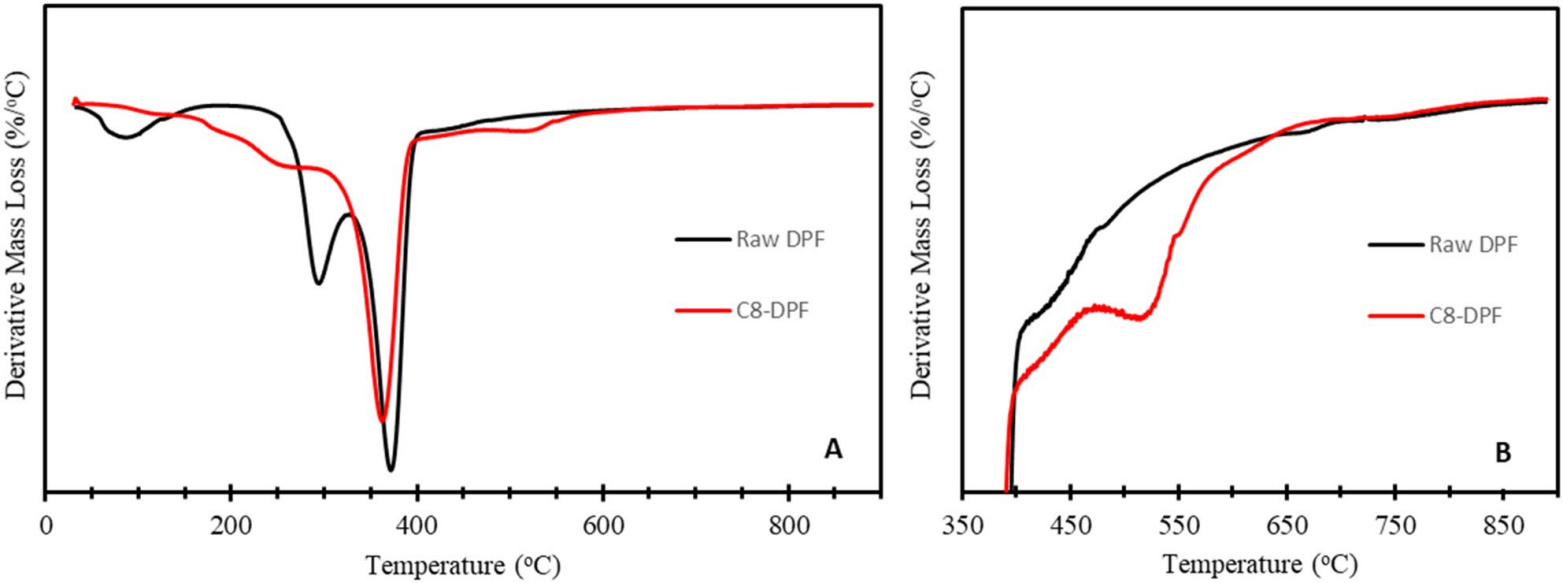
DTGA thermograms of raw DPF and C8-DPF.

The TGA thermograms for raw and C8 treated DPF samples are shown in Fig. 3. To more clearly show the nature of thermal degradation, the first derivative of mass losses are potted against temperature. Figure 3A, the derivative curves of raw DPF and C8 treated DPF, showed three major mass losses in the temperature range of 25–400 °C. The first mass loss was associated with the removal of physically adsorbed water. The other two major degradations occur between 200 and 400 °C, related to the degradation of hemicellulose and lignin^28,29^. After modification of C8 on DPF, a decrease in mass losses of hemicellulose and lignin were observed on the C8-DPF compared to raw DPF. It indicates C8 silane treatment can remove hemicellulose and lignin or non-crystalline cellulose present in the fiber^30^. A significant broad mass loss occurred between 375 and 750 °C in raw and C8 treated DPF, as shown in Fig. 3B. For raw DPF, this broad mass loss was attributed to the degradation of cellulosic and other non-cellulosic materials present in the DPF^28,29^. The decomposition of C8-DPF gave a well-resolved peak around 520 °C, in addition to the broad mass loss, as shown in Fig. 3B. According to the literature, the pronounced mass loss in the temperature range 450–600 °C was attributed to the decomposition of the hydrocarbon chain of C8^31^. TGA thermograms confirm raw DPFs were successfully modified with the C8 silane coupling agent.

### Fourier transform infrared (FTIR) spectroscopy

**Figure 4:**
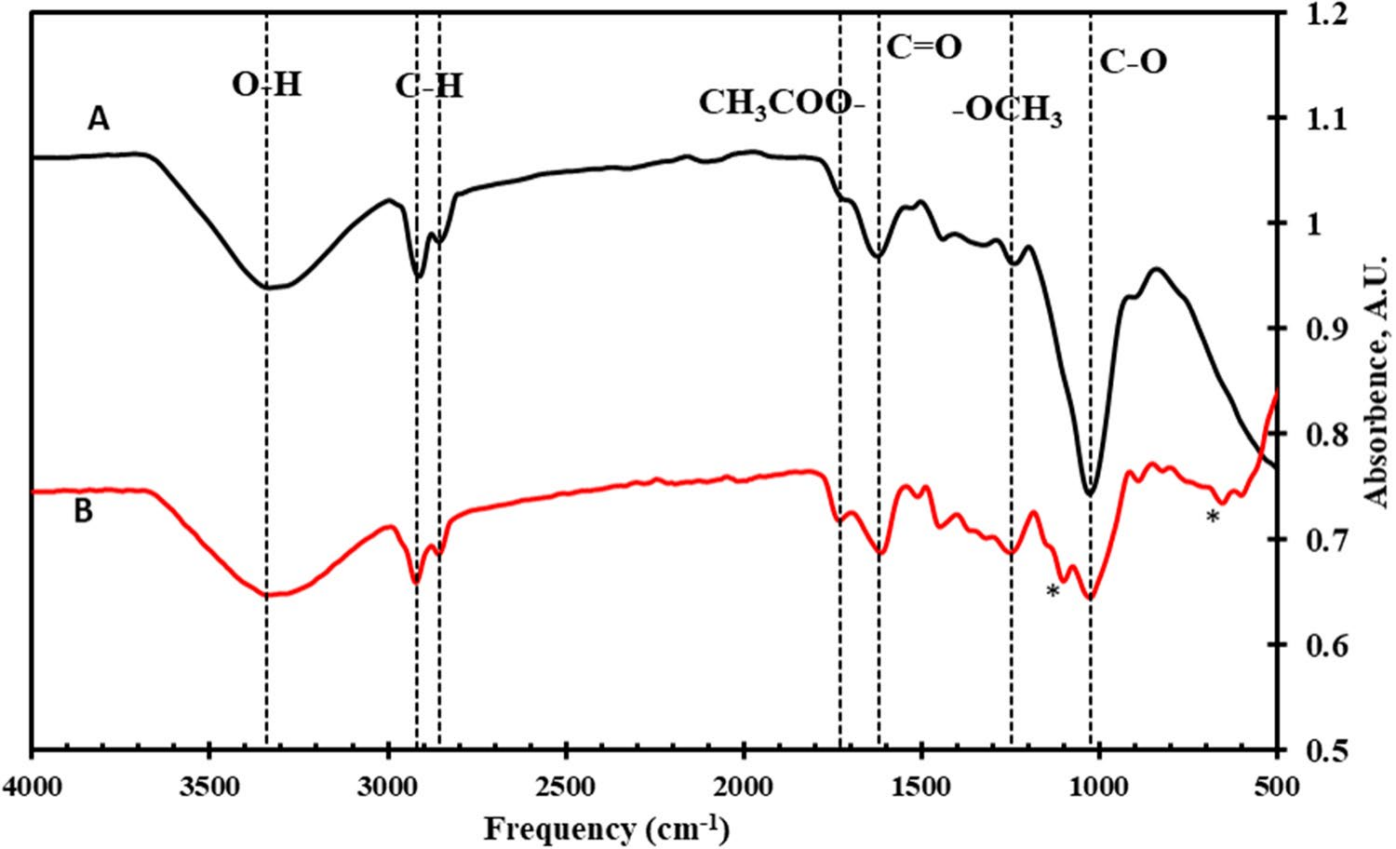
FTIR spectra for (**A**) raw DPF and (**B**) C8-DPF. Additional peaks appear after the C8 modification indicated with the * symbol.

The structural changes in the fiber surface before and after treatment were investigated using FTIR spectroscopy to establish the chemical efficiency of silane treatments. FTIR spectra of the raw and C8-DPF are shown in Fig. 4. The common peak positions for raw and C8-DPF are indicated with the dotted line. The broad peak ranging from 3660 to 2990 cm^−1^ was because of hydroxyl groups stretching vibration present in cellulose, hemicellulose, and lignin. The vibration peaks at 2919 cm^−1^ and 2854 cm^−1^ revealed asymmetric and symmetric CH_2_ stretching in cellulose/hemicellulose, respectively. The peaks at 1730 cm^−1^, 1620 cm^−1^, 1245 cm^−1^ and 1023 cm^−1^ are corresponded to ester carbonyl group stretching, C=O stretching in carboxylic acid in hemicellulose, O-CH_3_ stretching in lignin and C–O stretching vibration, respectively, for both raw and C8-DPF^11,12,32^. After C8 silane treatment, new absorption bands are appeared in the region, between 1200 to 500 cm^−1^, which are specific for silane coupling agents. Indeed, two new bands emerge at 1100 cm^−1^ and 670 cm^−1^, which are caused mainly by the vibration of Si–O-cellulose and Si–O–Si on the fiber, which is shown in Fig. 4 shows the * symbol^12,33,34^. The FTIR results demonstrated that the C8 silane coupling agent chemically treats raw DPF surfaces.

### Scanning electron microscopy (SEM)

**Figure 5:**
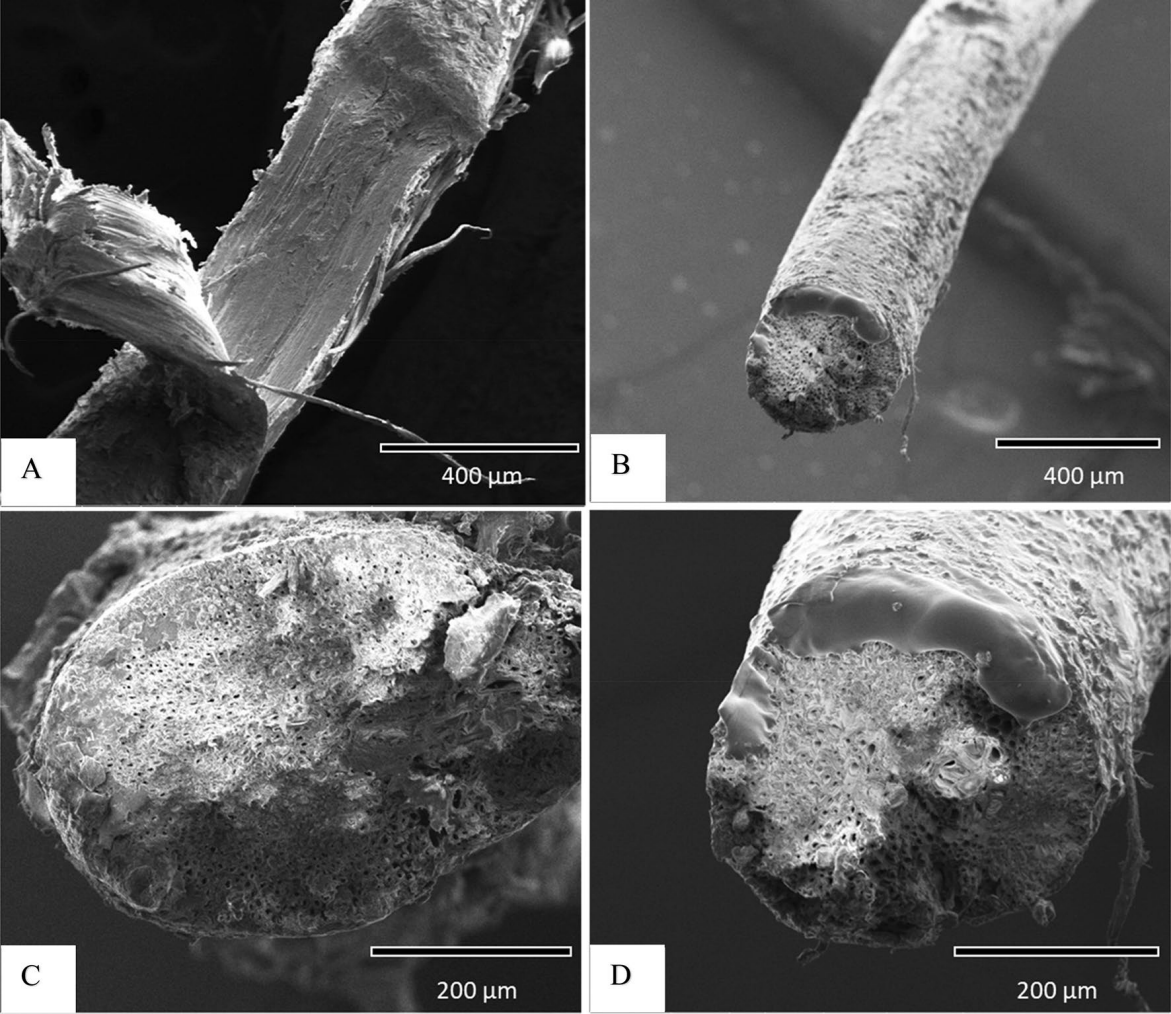
SEM of the longitudinal surfaces of (**A**) raw DPF and (**B**) C8-DPF, the cross sections of (**C**) raw DPF and (**D**) C8-DPF.

SEM is a powerful tool for studying the surface morphology of fibers. Figure 5 shows the relevant SEM images of the raw and C8 modified DPF. Figure 5A,B show the SEM images of the longitudinal surface of raw and C8-DPF. DPF has a cylindrical shape both with and without treatment, as shown in Fig. 5A,B. Silane treatment did not damage the shape of the fiber. When comparing raw DPF to C8 treated fiber, the diameter of the silane treated fiber is smaller. The removal of hemicellulose and lignin from the fibers causes the diameter to shrink. The findings are consistent with recent experimental data on oil palm fiber by Yousif et al.^35^, kenaf fiber by Chin and Yousif et al.^36^ and hemp fiber by Sawpan et al.^37^. As illustrated in Fig. 5A,B, the raw DPF surface seems rougher, whereas C8-DPF appears to develop a smoother surface due to the filling up of the spaces by silane treatment. Figure 5C,D show SEM micrographs of raw and C8-DPF in the cross sections, respectively. With C8 treatment, fiber pores are increased due to the removal of hemicellulose and lignin.

### Contact angle analysis

**Figure 6:**
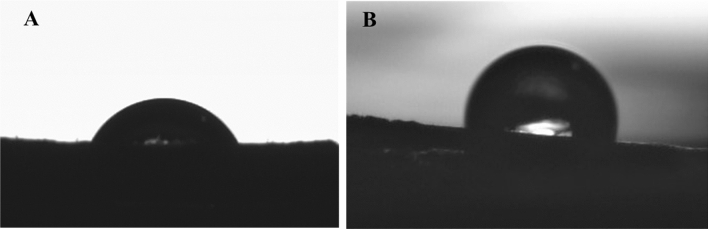
The contact angles of (**A**) raw DPF and (**B**) C8-DPF.

Hydrophobicity of the surface was measured using contact angle measurements. Water contact angles of raw and C8 treated DPF are shown in Fig. 6. The raw DPF had a contact angle of 66.8° ± 3°. Free hydroxyl groups present on hemicellulose and lignin makes raw DPF hydrophilic^38^. The hydrophobicity of the raw DPF was enhanced to 116° ± 24° with modification of the DPF with the C8 silane coupling agent. Introducing low surface energy material improved the hydrophobicity of the material^39^.

### X-ray diffraction (XRD)

**Figure 7:**
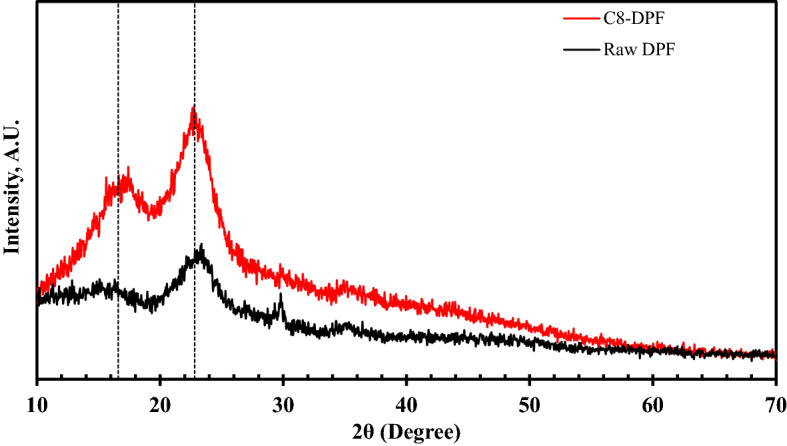
XRD patterns for raw and C8 treated DPF.

XRD analysis is most commonly used to determine the crystallinity and physical structure of the sample after the modification. Figure 7 exhibits the XRD pattern for raw and C8 treated DPF. The diffractogram of raw and C8 treated DPF shows two peaks commonly seen in DPF^8,17,19^. The first peak at 16.6° corresponding to the 101 planes represents the presence of amorphous constituents of cellulose, hemicellulose and lignin. The second peak, 23°, corresponds to the 200 plane represents the presence of α-cellulose^8,17,19^. The experimental results reveal that during surface treatments with C8 silane, there is no structural transformation from cellulose.

Table 1 shows the crystallinity index (CI) of the raw and C8 treated DPF, calculated based on the Eq. (1). The calculated CI for the raw and C8 treated DPF was 31% and 41%, respectively. The rise in CI was observed due to the effective removal of amorphous cellulose from the fiber surface. These results are supported by the SEM micrographs, shown in Fig. 5C,D.

**Table 1:** Crystallinity index of the raw and C8 treated DPF.

Type of sample	Crystallinity index (%)
Raw DPF	31
C8-DPF	41

## Conclusions

In this research, DPF was treated with the C8 silane coupling agent and modification was able to confirm through TGA, FTIR, SEM and XRD results. With treatment of C8 thermal stability increased compared to raw DPF as per thermograms. Alkylsilane treatment can remove the non-crystalline cellulose (hemicellulose and lignin) on DPF, confirmed by decreasing mass loss of non-crystalline cellulose and increasing fiber pore size and crystallinity index, respectively, in TGA, SEM and XRD results. With the incorporation of the silane coupling agent, the hydrophilicity of the fiber converted into hydrophobic due to the lower surface energy by C8 silane coupling agent. The results indicated that alkylsilane-treated DPFs were a suitable reinforcing substitute for hydrophobic polymer composite.

## Data Availability

All data generated or analysed during this study are included in this published article. The datasets used and/or analysed during the current study available from the corresponding author on reasonable request.
